# The prediction of impacted versus spontaneously erupted mandibular third molars

**DOI:** 10.1186/s40510-021-00376-2

**Published:** 2021-09-27

**Authors:** Amanda Silva De Sousa, José Valladares - Neto, David Normando

**Affiliations:** 1 Department of Orthodontics, Faculty of Dentistry, Federal University of Pará, Belém, Brazil; 2 Department of Orthodontics, Federal University of Goiás, Goiânia, Brazil; 3 Department of Orthodontics, Federal University of Pará, Avenida Pedro Àlvares Cabral, 880, Apto 2500, Bairro Umarizal, Belém, Pará CEP 66050400 Brazil

**Keywords:** Third molar, Tooth extraction, Orthodontist, Oral surgery

## Abstract

**Background:**

To evaluate the predictive capacity of orthodontists and oral maxillofacial surgeons (OMFSs) in anticipating the process of impaction or eruption of lower third molars (L3Ms) through the examination of serial panoramic radiographs.

**Methods:**

Sixty-eight lower third molars (L3Ms) were analyzed in 34 orthodontically treated patients without extraction. Twenty-seven OMFSs and 27 orthodontists were randomized in order to analyze the radiographs. Initially, the evaluators issued the prognosis for the L3Ms in XR1, a posterior for the XR1 + XR2. Concordance of the diagnosis was examined using Kappa statistics, and the differences between the groups of evaluators were examined using the chi-square test at *p*<0.05.

**Results:**

When examining XR1 in cases where the teeth erupted spontaneously, the prognostic accuracy rate for OMFSs and orthodontists was similar, 63 and 65.7%, respectively (*p*=0.19). When evaluating XR1 + XR2, the accuracy among orthodontists (60%) was similar to that reported for XR1 (*p*=0.19), while OMFSs presented a reduction in the accuracy (55.3%, *p*<0.0001). When the L3Ms remained impacted, accuracy in XR1 was lower than in spontaneously erupting L3Ms, although similar between OMFSs (50.1%) and orthodontists (49.1%). Furthermore, for impacted L3Ms, when examining XR1 + XR2, the OMFSs presented a significant higher accuracy (71.8%, *p* <0.0001).

**Conclusions:**

Orthodontists and OMFSs seem unable to predict spontaneous eruption or impaction of the lower third molars from single or longitudinal x-rays. When adding a second longitudinal x-ray, orthodontists and more significantly OMFSs tend to indicate more extractions.

## Introduction

The third molars, among all teeth, are the ones with the highest frequency of impaction, often being asymptomatic throughout life [1]. Therefore, diagnosing the need for surgical removal or preservation of third molars is a recurring question [2, 3], although in asymptomatic cases the treatment is the careful monitoring.

Prophylactic extraction of third molars, even in the absence of local disease, is one of the most common surgical procedures [4]. In this context, the critical evaluation of the literature reveals that exodontia is indicated without a clearly defined criteria [5], this is because approximately 75% of individuals receiving regular dental care have their third molars removed [6]. However, when a clinical practice guideline is applied, there is a drastic reduction in the number of indications for extractions in simulated cases [7].

The decision to extract third molars should be made according to well-defined criteria [8]; thus, it is important to instruct professionals to recognize the benefit of the early removal of the third molars in cases where the extraction is really well indicated [7, 9]. On the other hand, any recommendation for the removal of asymptomatic third molars in order to avoid future complications should be questioned [8]. Clinical decisions seem to be centered on a preference for each specialty, although the professional must base his decision to extract or not to extract the third molars based on the most recent scientific information and it must be individualized to each patient [10]. If there is adequate space for the eruption of the third molars, all efforts should be made to place these teeth into functional occlusion [11].

The most conservative approach is to carefully monitor asymptomatic third molars. This approach is based mainly in the absence of scientific evidence to justify prophylactic extraction. Monitoring should be performed every 2 years up to at least the age of 18. The serial analysis of panoramic radiographs, a method widely used for clinical monitoring of orthodontic patients, might be able to increase the accuracy of this prediction [12].

Whenever indicating extraction of third molars, dentists, should have a justifiable reason, taking into account future treatment planning from an orthodontic, surgical, periodontal, and/or prosthetic point of view [12].

The literature reveals a great conflict between what is published by scientific evidence and clinical practice. Much of this excitement is related to the difficulty of having a prognosis in front of a developing third molar. Orthodontists and oral maxillofacial surgeons do not seem to be able to predict the spontaneous eruption of the lower third molars by examining a single panoramic radiograph that has spontaneously erupted [10], or even through serial radiographs in cases of spontaneous eruption. However, in spite of this knowledge, there is no information about the accuracy of a prognostic when the L3Ms remain impacted.

The decision to preserve or remove third molars remains unclear for orthodontists and surgeons, as these professionals’ motives for the indication seem to be different. A systematic review demonstrated the paucity of consistent articles and inadequate scientific evidence that could allow dentists to make decisions about reliable indications for prophylactic third molar extraction. And, consequently, orthodontists and surgeons could obtain a faithful judgment capable of predicting spontaneous eruption versus impaction, when determining which cases should be followed or not [6]. In this sense, the objective of this study is to evaluate the ability of oral maxillofacial surgeons (OMFSs) and orthodontists in predicting the spontaneous impaction or spontaneous eruption of mandibular third molars by serial panoramic radiographs, the method most used by clinicians.

## Material and methods

This study was approved by the Research Ethics Committee. The Free and Informed Consent Term (FICT) was signed by all the dental surgeons participating in the research, in addition to the Term of Commitment of Database Use (TCDU) by the orthodontist who provided the panoramic radiographs of his patients.

**Fig. 1:**
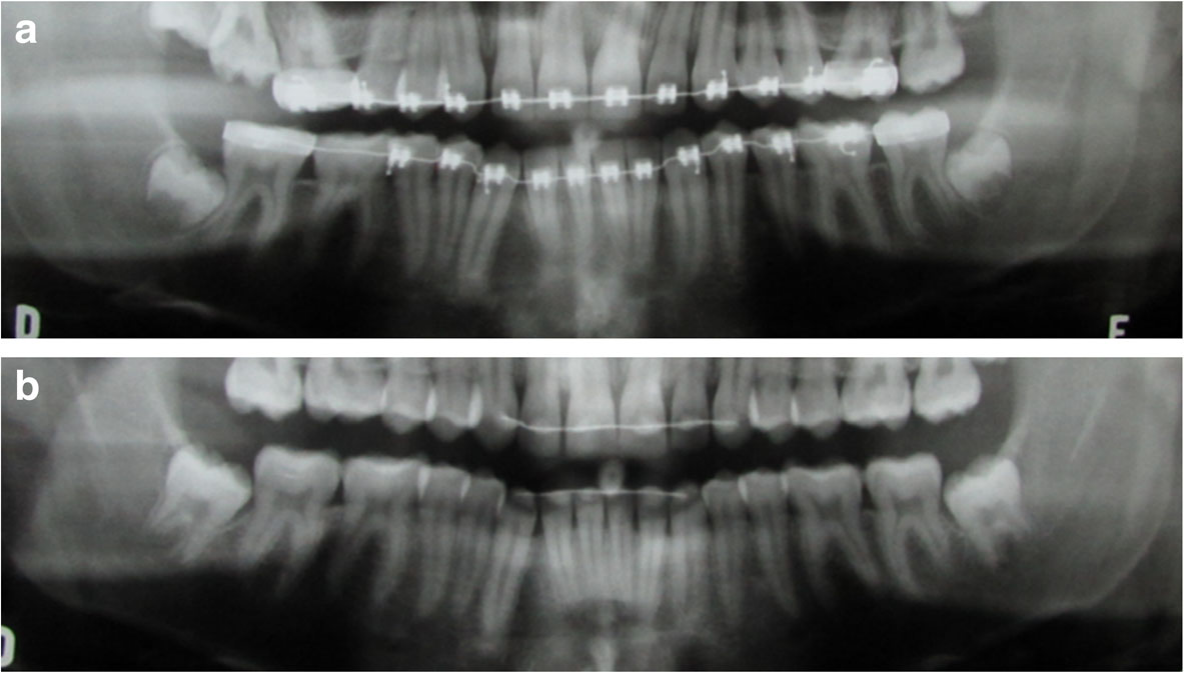
Panoramic radiograph at the end of orthodontic treatment (XR1) of patient # 21 (positive control group) at 15 years and 7 months of age (**A**), at age 18 and 4 months in XR2 (**B**)

The sample included 34 patients, whose panoramic radiographs, two for each patient, included 68 L3Ms, were retrospectively selected from patients who had undergone orthodontic treatment without dental extractions (and mechanics involving molar distalisation, Fig. 1A) and obtained from clinical records belonging to a single orthodontist in private practice. Eighteen males and 16 females were evaluated. All patients had a second follow-up radiograph, which was taken on average, 2 years later (XR2, Fig. 1B). Only patients with both L3Ms were included and subjects with tooth loss, agenesis, syndromes and/or cleft lip and palate were previously excluded. All patients included had a third x-ray to confirm the eruption or impaction of the lower third molar, which was not presented to the evaluators.

**Fig. 2:**
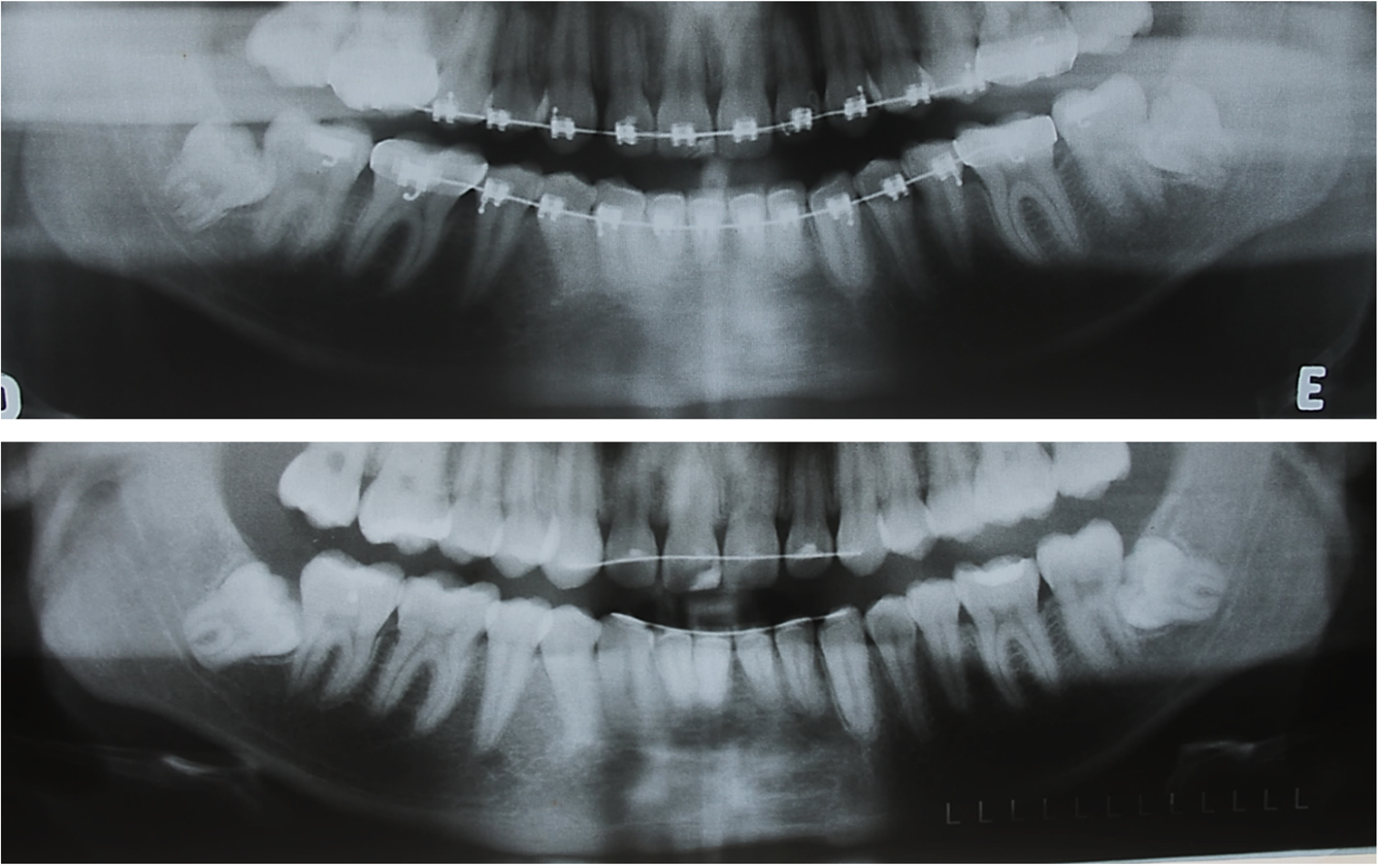
Panoramic radiograph at the end of orthodontic treatment (XR1) of patient # 17 (impacted group) at 16 years and 0 months of age (**A**) and at 20 years and 1 month in XR2 (**B**)

The mean age was 14.2 years old in XR1 (in the end of the orthodontic treatment) and 17.1 years old in XR2 (15–20.1 years). On XR1, the L3Ms were, in general, in stage six of Nolla of root formation, between 13 and 16 years old (Fig. 1A). L3Ms were followed until spontaneous eruption, without symptomatology (*n* = 44) or definitive impaction (*n* = 24, Fig. 2), where the mean age was 23.5 years (20.1–27 years).

Pictures from the radiographs were obtained with digital camera (Canon EOS Digital Rebel, Kiss X5, EF-S 18-55; Inc, Tokyo, Japan). The images were digitally cut out in a standardized way in order to show the region of the lower third molars and anatomical structures of the mandible, such as the lower border, mandibular angle, and the ascending ramus (Fig. 1A, B).

The radiographic images were randomly mounted using the Microsoft Power Point 2010 program (Microsoft, Redmond, USA), including age and sex, and presented for two groups of specialists. Twenty seven oral and OMFSs and 27 orthodontists were randomly assigned from a list of specialists. They were asked to predict the prognosis for the 68 lower third molars, bilaterally. Two OMFSs refused to participate in the study; however, there were no recruits for two more OMFSs and a new randomization was performed.

The total number of professionals enrolled in this study, 54 orthodontists and OMFSs, was based on a previous study, which was shown to have enough power to detect intergroup differences [10].

Firstly, a panoramic radiograph corresponding to the end of the orthodontic treatment (XR1) was presented to the evaluators (Fig. 1A), they were then asked for the prognosis of spontaneous eruption or impaction for both the L3Ms. The questionnaire included the following question, “After evaluation of the lower third molars, what would be your clinical approach?”. The answers were (1) extraction, (2) monitoring, and (3) other. Then, the evaluators examined the second radiograph together with the first one (XR1+2, Fig. 1B). When “extraction” was the was the chosen option by the respondent, he/she indicated at least one of the following reason: “(1) presence or potential risk to develop a pathology, (2) risk of resorption of the second molar, (3) can lead to dental crowding, (4) caries risk, (5) tooth impacted or with risk of impaction, and (6) other. There was no time limit for the respondents when analyzing.

To evaluate the error of the method, reliability was performed using four of the tooth images in two of the patients (# 22 and # 26), and this was re-evaluated by all 54 examiners. Statistical analysis, with a significance level of 5%, was performed using the software BioEstat 5.3 (Sustainable Development Institute Mamirauá, Belém, Pará, Brazil, 2011). Concordance of intra- and inter-examiner responses was examined using the Kappa test. In order to verify intra and inter-group differences in the answers to the questions among the professionals, the chi-square test was used.

## Results

For the initial X-ray examination (XR1), intra-examiner agreement was considered moderate for orthodontists (Kappa=0.46) and for OMFS (Kappa=0.47, Table 1). In the subsequent analysis, in which a second serial panoramic radiograph of the same patient (XR1+2) was examined together with XR1, a significant increase in agreement was observed for orthodontists (Kappa=0.65) and OMFS (Kappa=0.67).

**Table 1:** Intra-examiner concordance of oralmaxillofacial surgeons (OMFS) and orthodontists (ORTHO) when examined one (XR1) or two serial (XR1 + XR2) radiographs

	XR1				XR1 +2			
	ORTHO		OMFS		ORTHO		OMFS	
	CRM	Exo	CRM	Exo	CRM	Exo	CRM	Exo
CRM	72	10	53	11	62	11	37	9
Exo	11	15	16	28	6	29	8	54
Kappa	0.46		0.47		0.65		0.67	
p value	<0.0001		<0.0001		<0.0001		<0.0001	

*CRM* clinical and radiographic monitoring, *Exo* exodontia

In the analysis of the first radiograph (XR1), the prognostic accuracy for all lower third molars, erupted spontaneously or impacted, was not different between OMFSs (58.3%) and orthodontists (59.9%, Table 2). When adding a second serial radiograph (XR1+2), accuracy was significantly different between OMFSs (54.3%) and orthodontists (60.2%, *p*<0.0001) (Table 2).

**Table 2:** Frequency and difference of the responses indicated by oralmaxillofacial surgeons (OMFS) and orthodontists (ORTHO) when evaluating all lower third molars, erupted spontaneously or impacted (n = 68), after analysis of one (XR1) or two serial radiographs (XR1 + 2)

	OMFS			ORTHO		
	XR1			XR1		
XR1+2	**Correct**	**Error**	**Total**	**Correct**	**Error**	**Total**
Correct	743 (40.5%)	254 (13.8%)	997 (54.3%)	797 (43.4%)	309 (16.8%)	1106 (60.2%)
Error	327 (17.8%)	512 (27.9%)	839 (45.7%)	303 (16.5%)	427 (23.3%)	730 (39.8%)
Total	1070 (58.3%)	766 (41.7%)	1836 (100%)	1100 (59.9%)	736 (40.1%)	1836 (100%)
Kappa	0.35 (p-valor <0.01)			0.30 (p-valor <0.01)		
X^2^ XR1 OMFS vs Ortho	<0.0001			<0.01		
X^2^ XR1+2 OMFS vs Ortho	<0.0001			<0.0001		

The concordance of the prognosis indicated when the professional examined only the first radiograph (XR1) compared to that obtained from XR1+2 was considered weak for OMFSs (Kappa=0.35) and for the orthodontists (Kappa=0.30).

In cases where the lower third molars erupted spontaneously (*n*=44), when examining the first radiograph (XR1), the prognosis accuracy for OMFSs and orthodontists was similar (*p*=0.198), 63 and 65.7%, respectively (Table 3). When examining XR1+2, accuracy among orthodontists (60%) was similar to that reported for XR1 (*p*=0.198), while OMFSs showed a significant lower accuracy (44.7%, *p*<0.0001). The concordance comparing XR1 prognostic to that obtained with two serial radiographs (XR1+2) was weak for OMFSs (Kappa=0.07) and for orthodontists (Kappa=0.25).

**Table 3:** Frequency and difference (X^2^) of the responses indicated by oralmaxillofacial surgeons (OMFS) and orthodontists (ORTHO) when evaluating spontaneously erupted lower third molars (n=44) after analysis of one (XR1) or two serial radiographs (XR1 + 2)

	OMFS		ORTHO	
	XR1	XR1+2	XR1	XR1+2
Correct	749 (63%)	531 (44.7%)	780 (65.7%)	713 (60%)
Error	439 (37%)	657 (55.3%)	408 (34.3%)	475 (40%)
Total	1188 (100%)	1188 (100%)	1188 (100%)	1188 (100%)
Kappa	0.077 (p<0.0001)		0.256 (p<0.0001)	
X^2^ XR1 OMFS vs ORTHO	0.198			
X^2^ XR1+2 OMFS vs ORTHO	<0.0001			

When lower third molars were impacted (n=24), accuracy was lower than teeth that erupted spontaneously on XR1, although similar between OMFSs and Orthodontists, 50.1% and 49.1%, respectively (*p*=0.73, Table 4). In XR1+2, the OMFSs presented a considerable increase in accuracy (71.8%, *p*<0.0001). The percentage of accuracy prognostic among orthodontists also increased, but was lower compared to OMFSs (59.8%, *p*<0.0001). The concordance coefficient between XR1 evaluation and two serial radiographs (XR1+2) was weak for OMFSs (Kappa=0.21) and for orthodontists (Kappa=0.08).

**Table 4:** Frequency and difference (X^2^) of the responses indicated by oralmaxillofacial surgeons (OMFS) and orthodontists (ORTHO) when evaluating impacted lower third molars (n=24) after analysis of one (XR1) or two serial radiographs (XR1+2)

	OMFS		ORTO	
	XR1	XR1+2	XR1	XR1+2
Correct	325 (50.1%)	465 (71.8%)	318 (49.1%)	387 (59.8%)
Error	323 (49.9%)	183 (28.2%)	330 (50.9%)	261 (40.2%)
Total	648 (100%)	648 (100%)	648 (100%)	648 (100%)
Kappa	0.219 (p<0.0001)		0.088 (p=0.0007)	
X^2^XR1 OMFS vs ORTHO	0.738			
X^2^ XR1+2 OMFS vs ORTHO	<0.0001			

After examining only the first panoramic radiograph (XR1), the OMFSs (52.74%) and orthodontists (50.67%) indicated the option “impacted or impacted tooth,” as the most frequent justification for the exodontia of L3Ms (Table 5). When evaluating XR1+2, OMFSs and orthodontists justified the exodontia mainly due to “impaction” (53.88 and 64.62%, respectively). No orthodontist considered the “crowding” option, either when evaluating XR1 or XR1+2, as justification for the extraction of L3Ms. Among the OMFSs, this justification was pointed out by 2.08% of the professionals in XR1 and 1.14% in XR1+2.

**Table 5:** Frequency of the justifications for the extraction of the lower third molars when orthodontists (ORTHO) and oralmaxillofacial surgeons (OMFS) examined one (XR1) or two serial (XR1+2) radiographs

Justifications	XR1 (n=34)		XR1+2 (n=34)	
	OMFS (n=27)	ORTHO (n=27)	OMFS (n=27)	ORTHO (n=27)
Pathology	172 (15.48%)	13 (1.35%)	260 (17.38%)	21 (1.98%)
Resorption	210 (18.90%)	314 (32.48%)	202 (13.50%)	167 (15.75%)
Crowding	23 (2.08%)	0	17 (1.14%)	0
Caries	79 (7.11%)	140 (14.47%)	158 (10.56%)	173 (16.32%)
Impaction	586 (52.74%)	490 (50.67%)	806 (53.88%)	685 (64.62%)
Others	41 (3.69%)	10 (1.03%)	53 (3.54%)	14 (1.33%)
Total	1111	967	1496	1060

## Discussion

The classification of third molars regarding their position leads to different treatment behaviors among professionals, especially when they are asymptomatic and without associated pathologies [3]. Some professionals are influenced by the strong preventive philosophy in dentistry, while others are influenced by the number of pathologies associated with mandibular third molars [13].

Overall, when only one panoramic radiograph at the end of orthodontic treatment was examined (XR1), the prognostic accuracy for lower third molars eruption was similar to the chance of accuracy by chance, even for orthodontists (59.9%) or OMFSs (58.3%). When adding another serial radiograph obtained 2 years later (XR1+XR2), no significant difference was observed among orthodontists (Table 2); however, among OMFSs, there was a lower accuracy in cases of spontaneous eruption.

When analyzing spontaneously erupted lower third molars (*n*=44), it was observed that the prognostic accuracy rate for XR1 was similar (*p*=0.19) for both groups of specialists (63% for OMFSs and 65.7% for orthodontists, (Table 3). However, when a second serial radiograph was included, the accuracy was worsen for OMFSs (55.3%). OMFS tends to indicate more extraction of teeth that will erupt spontaneously. For orthodontists, the accuracy was not improved when a second serial X-ray is evaluated.

In cases where lower third molars were impacted (*n*=24), the prognostic accuracy was similar to the probability of a right choice by chance for OMFSs (50.1%) and orthodontists (49.1%). When XR1+2 are examined, OMFSs improved significantly their accuracy (71.8%, *p*<0.0001). Orthodontists also indicated more extraction, but to a lesser extent than OMFSs (59.8%, *p*<0.0001). Thus, these findings confirm that there is a bias observed among specialists, since decision-making inevitably implies an element of subjectivity and individual variations can be expected [13]. Surgeons tended to indicate more extractions than orthodontists, regardless of whether the third molar will erupt spontaneously or will impact.

The concordance of the responses pointed out by orthodontists and OMFSs on the approach adopted for the third molars impacted or spontaneously erupted (*n* = 68), in general, was poorer for both group of evaluators when only one radiograph was examined when compared to the diagnosis obtained with the two radiographs (XR1+2) (*p*<0.0001). This agreement differs from that obtained in previous studies [10], where, through the analysis of a single panoramic radiograph, an excellent agreement was observed. Because they are different clinical cases, it is likely that the level of agreement depends fundamentally on the case analyzed.

The risk of impaction was the main motivator that led the specialists to indicate extractions of the third molar in our findings (Table 5), in contrast to the justifications found in a previous study [10], which pointed out that the risk of developing diseases was the key motivator leading orthodontists and OMFSs to indicate third molar extractions in their findings. This data may be associated with the eruption pathway of the lower third molars with their angulation directed to mesial that may cause a more intimate contact with the adjacent tooth. Advanced root development and the end of the retromolar space growth are widely reported as determinants of the impaction of the third molar; these have caused an increase in the indication of the impaction as a risk factor.

The influence of third molar eruption on the anterior crowding of the lower incisors has been studied [14]. However, this association is not significant and does not justify removal of the third molars [6, 15]. Systematic reviews contraindicate the prophylactic removal of third molars in order to avoid late crowding in the anterior region of the mandible [11], since none of the orthodontists, when evaluating only one or both panoramic radiographs, cited the crowding of lower incisors as justification for extraction of the lower third molars (Table 5). This is contrary to the results of previous studies [16], in which many orthodontists (30.3%) and OMFSs (55%) report that L3Ms are capable of producing crowding.

This research used a more comprehensive and known radiographic view in the clinical area to evaluate the lower third molars, the panoramic radiograph. The advantage of these radiographs is a complete overview of the dentition in relation to the stage of dental development, number of teeth, dental malformations, eruption, and resorption processes [3, 17]. The problem in evaluating the eruption of the third molar on panoramic radiographs is the difficulty of accurately evaluating skeletal parameters; this method does not replace the clinical evaluation of the patient. Thus, radiographic image formation is directly affected by the shape of the image layer, which varies among different panoramic X-rays. The evaluation parameters of the panoramic radiograph, whether taken at the end of the orthodontic treatment or as a long-term evaluation, do not offer as many requirements to confirm what will occur with the lower third molars, according to the professionals interviewed.

A previous study [18] reported no differences between panoramic and multiplanar CBCT images regarding the assessment of prognosis of third molars. However, there were significant differences in relation to the professional decision regarding the prognosis of these teeth, where OMFS have indicated more extractions than orthodontists.

The likelihood of impacted third molars causing future pathological changes may be exaggerated [7]. Many impacted or untreated third molars erupt normally and never cause clinically important problems. In addition, third molar surgery is not risk free; complications and suffering after surgery should be considered. Therefore, prophylactic removal should be performed if there is good evidence that it will benefit the patient. Thus, computed tomography is considered a more accurate technique to evaluate the involvement of anatomical structures, such as the mandibular canal, with the lower third molars [3].

The mean age of the patients in our study was 14.2 years old on the first panoramic radiograph and 17.1 years old on the second radiograph. The sample of patients in the control group of spontaneous eruption was larger and easier to acquire in comparison to the impaction group. This suggests that the third molars erupt more spontaneously in larger numbers than they are impacted.

Third molars become more upright until age 25 and can erupt normally, usually between 18 and 24 years of age. The final age for diagnosis of eruption or impaction is, on average, 21 years old [15]. The present study included third molars that had no clinical symptoms; therefore, the teeth examined represent a selective sample.

The retrospective nature of this research increases the possibility of bias and was a limiting factor in this study. This research has limited external validity, as only orthodontic patients were included in the sample and not all clinicians perform this number of radiographs for all their patients. The absence of sample size calculation also becomes a limiting factor in this research. The findings and conclusions of this study should be limited to patients with both mandibular molars present. Unilateral conditions may behave differently [2].

Since there is no reliable way to predict the risks of pathological changes related to impacted third molars, the most conservative strategy would be to monitor the life cycle of these elements through radiological monitoring at regular intervals [19]. On the other hand, when these teeth are involved with some painful symptomatology, there is a general consensus that extraction is necessary.

## Conclusion

Orthodontists and OMFSs seem unable to predict the spontaneous eruption or impaction of the lower third molars through a single panoramic radiograph taken at the end of orthodontic treatment in adolescents. By adding a second serial panoramic radiograph, orthodontists and in particular, OMFSs, tend to indicate more extractions. This increases the percentage of error when the L3Ms erupted spontaneously and reduces it when the L3Ms become impacted.

The analysis of the replicated cases revealed a higher concordance of the responses when the two serial radiographs were examined by both groups of examiners. This seems to suggest that the higher the stage of development of the third molar; the greater concordance will be observed.

## Data Availability

All data generated or analyzed during this study are included in this published article.

## Abbreviations

OMFSs: Oral maxillofacial surgeons
L3Ms: Lower third molars
XR: X-ray
FICT: Free and Informed Consent Term
TCDU: Term of Commitment of Database Use
